# Secondary brain metastases of Ewing's sarcoma presenting with collapse after 6 years of complete remission

**DOI:** 10.1002/ccr3.3583

**Published:** 2020-11-24

**Authors:** Jian Zi Poh

**Affiliations:** ^1^ Trauma and Orthopaedics Department Scunthorpe General Hospital Scunthorpe UK

**Keywords:** brain tumor, computed tomography, Ewing's sarcoma, metastases

## Abstract

Routine brain imaging with MRI and long‐term follow‐up of Ewing's sarcoma could be the way to move forward by increasing our understanding in this area, as well as improving treatment and long‐term outcome for patients.

## Graphical abstract

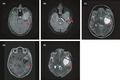

## 1 INTRODUCTION

A 23‐year‐old gentleman came to Emergency Department with first episode of sudden collapse. He had a right pelvic Ewing's sarcoma surgically resected 6 years ago. MRI of the head demonstrated a lesion in right temporal lobe, extending into infratemporal fossa. He was referred to oncology for further management with chemotherapy.

Ewing's sarcoma is a childhood tumor which is rarely seen, occurring in 2.93 children per 1 000 000, more common in male and hardly understood by many clinicians. The definite diagnosis is of Ewing's sarcoma is usually made in tertiary hospital via biopsy and microscopic evaluation of tumor cells and molecular analysis. Ewing's sarcoma usually requires treatment from different specialties including pediatric or adult oncologist, orthopedic surgeons, and other specialists depending on tumor site. Management of Ewing's sarcoma varies greatly among different patients but includes chemotherapy, radiotherapy, and surgical resection. Most of the brain metastases from Ewing's sarcoma usually occur in the range of 20‐30 months from the time of diagnosis of primary tumor. This case report describes the rare presentation of a gentleman who develops single secondary brain metastases of Ewing's sarcoma after 6 years of complete remission.

## 2 CASE HISTORY

A 23‐year‐old Caucasian gentleman was brought into the Emergency Department after an episode of collapse with loss of consciousness for 2 minutes. Up‐rolling of both eyeballs was seen throughout the episode.

He had been having 2 months history of intermittent pain around left temporomandibular area, not relieved by any analgesics. He had previous history of Ewing's sarcoma found in his right pelvis on May 2013. It was surgically removed via hemipelvectomy on January 2014. Postoperatively, he was continued with 16 courses of chemotherapy and 25 sessions of radiotherapy in total. He was then followed up by oncology department every 6 months for 4 year, followed by annually for 2 years. A chest and pelvic X‐ray was done before every appointment. He was discharged from follow‐up on Jan 2020 disease‐free. Apart from that, he had no other medical or surgical condition.

### 2.1 Examination

On physical examination, patient was alert and well orientated with Glasgow Coma Scale of 15/15. There was a small bruise to right side of temple with no laceration, possibly due to the collapse. No swelling or tenderness upon palpation was felt on the left side of the face. Upon cranial nerve examination, the only positive sign was a horizontal nystagmus seen in both eyes. There was also no abnormality noted in sensory and motor neurological examination of bilateral upper and lower limbs.

### 2.2 Investigations

Routine blood test including full blood count, urea and electrolyte, and C‐reactive protein was performed. No abnormality or raised inflammatory marker was noted.

Computed tomography scan of the head was requested. A space occupying lesion at the left temporal lobe extending through the skull base to the left masticator space was found. This warranted for a further contrast‐enhanced magnetic resonance imaging (MRI) of the head. An aggressive large mass lesion measuring 7 cm by 4 cm was seen in the left temporal location, eroding through the skull base, resulting in extracranial component in the infratemporal fossa. It had solid and cystic components with surrounding edema. Medially, the lesion was seen to extend up to the pterygoid plates and the posterior superior nasopharynx on the left side, and a resultant block of the Eustachian tube and a left mastoid effusion was seen. Midline shift to the right of 6mm as a result of mass effect was also noted. CT‐guided biopsy of the left superficial temporal fossa lesion was carried out. Presence of a CD99 + small round blue cell tumor and detection of an EWSR1 rearrangement at 22q12 in 84% of cells via FISH testing are consistent with diagnosis of recurrent Ewing's sarcoma.

**FIGURE 1:**
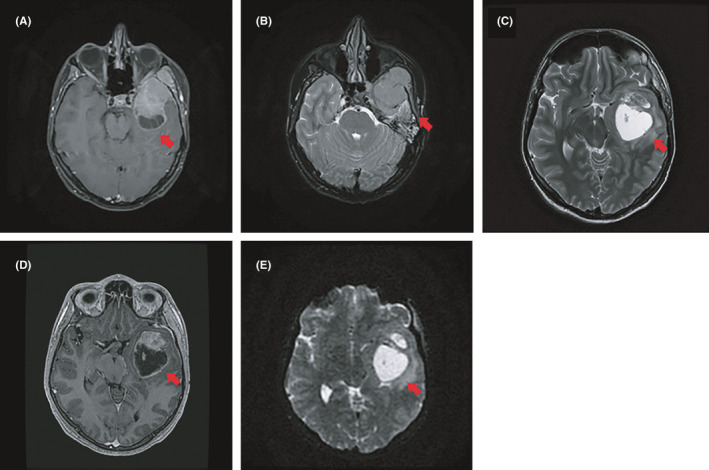
Axial MRI images A (T1W), B (T2W), C (T2 PROPELLER), D (FSPGR), and E (Diffusion weighted) demonstrating 7 cm by 4 cm lesion which consists of solid and cystic components with surrounding edema. Mass effect of the lesion causes midline shift of 6 mm to the right

**FIGURE 2:**
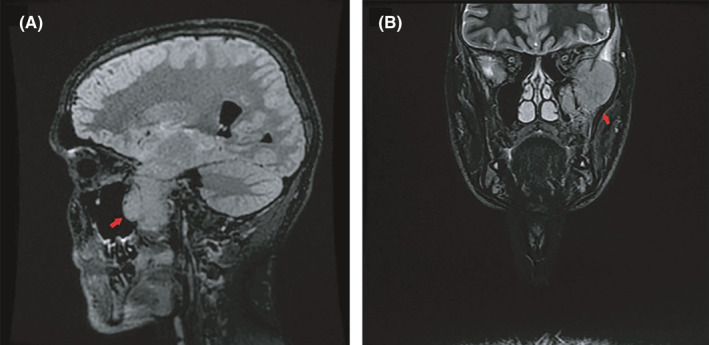
Sagittal (A) and coronal (B) MRI images demonstrating lesion in left temporal lobe eroding through base of skull with extracranial extension in the infratemporal fossa. Lesion extends up to pterygoid plates and posterior superior nasopharynx on the left side

After that, patient had a CT thorax, abdomen, and pelvis with contrast, showing no evidence of visceral metastases disease. He then proceeded to have a whole‐body SPECT and whole‐body PET FDG, showing single brain metastases with elevated activity in left skull base region and no evidence to suggest extracranial or bone metastases elsewhere Figures 1, 2.

### 2.3 Outcome and follow‐up

**FIGURE 3:**
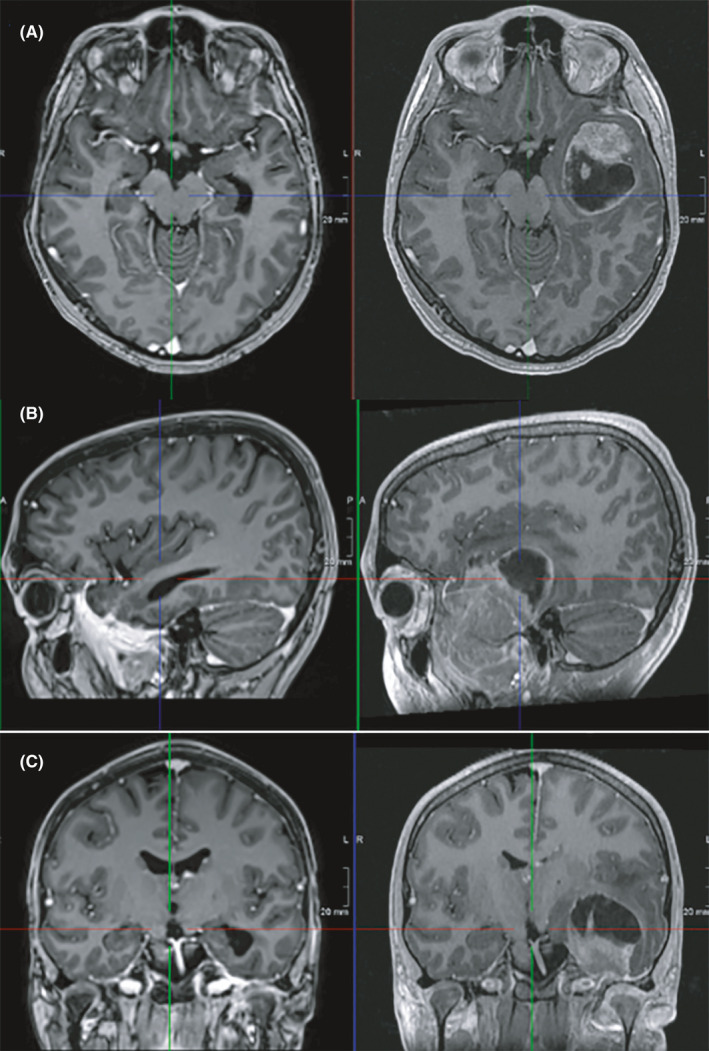
Dramatic regression of both intracranial and extracranial components of tumor is seen after chemotherapy. Comparison of images before chemotherapy (right) and after chemotherapy (left) are seen in A (axial view), B (sagittal view) and C (coronal view)

**FIGURE 4:**
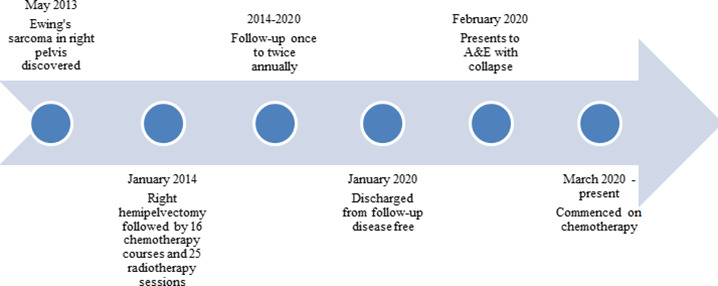
Figure illustrating chronological order of patient's disease and progression

The patient was then referred back to the initial oncology department for further management. At time of writing this report, the patient has had five courses of chemotherapy, ifosfamide, and etoposide (Doxifos) and had an MRI showing shrinkage of tumor by more than 50% after second course of chemotherapy. He will be continued with more courses of Doxifos and possible surgical resection Figures 3 and 4.

## 3 DISCUSSION

Ewing's sarcoma is a very rare type of bone or soft tissue tumor, predominantly affecting children, adolescents, and young adult. It is the however the second most common type of malignant bone tumors after osteosarcoma. The annual incidence of Ewing's sarcoma is around 1.8 per million for age 0‐14 and 3.7 per million for age 15‐24. The incidence rate is highest among the 15‐19 age group, which is 4.8 per million per year. It is also more commonly seen in male as compared to female with a ratio of 1.89:1.^1^ Ewing's sarcoma is more common among Caucasians as compared to the other races.^2^, ^3^, ^4^ Our patient, a 17‐year‐old Caucasian male at time of discovery, would fit into the category of highest incidence. Due to rarity of disease, the cause of Ewing's sarcoma is still unknown as most of the cases appear sporadically. Studies have shown that the t(11;22)(q24;q12) translocation is found in about 85% of Ewing's sarcoma, which leads to an EWS/FLI1 fusion gene but more studies have to be done to further understand the significance.^5^ It is in fact one of the most aggressive cancer with high metastatic potential.^6^

On first discovery of right pelvic Ewing's sarcoma 7 years ago, our patient had undergone a hemipelvectomy, coupled with a regimen of neo‐adjuvant chemotherapy, 25 sessions of radiotherapy, and a regimen of adjuvant chemotherapy. In fact, studies on pelvic Ewing's sarcoma have shown that surgery coupled with neo‐adjuvant chemotherapy and radiotherapy would produce better outcome as compared to radiotherapy coupled with neo‐adjuvant chemotherapy or radiotherapy alone.^7^, ^8^, ^9^, ^10^, ^11^, ^12^, ^13^ However, the results could be biased due to the fact that smaller sized tumors were managed surgically while large tumors were managed with neo‐adjuvant chemotherapy and radiotherapy. It is also seen that neo‐adjuvant chemotherapy would provide benefit on local control as compared to surgery with radiotherapy alone.^14^ Before surgical resection, our patient had neo‐adjuvant chemotherapy. He had undergone of 8 cycles of vincristine, ifosfamide, doxorubicin, and etoposide (VIDE) chemotherapy regime. VIDE chemotherapy is widely used across Europe in management of Ewing's sarcoma as it is considered the most effective treatment. It is also associated with low rate of adverse reactions. Our patient had an episode of neutropenia‐related fever during the course of VIDE, which is seen in 65.8% of the patients receiving this chemotherapy regimen.^15^ After 8 courses of VIDE, our patient had 25 courses of neo‐adjuvant radiotherapy, followed by surgical resection. He was then further managed with another 8 cycles of adjuvant chemotherapy. This chemotherapy regimen includes vincristine, actinomycin D or also known as dactinomycin, and ifosfamide (VAI). VAI is one of the most commonly used consolidation regimen. In fact, few studies and trial had been carried out to study on replacement of ifosfamide with cyclophosphamide due to the higher modifications needed for VAI; however, both of them were found to have similar efficacy.^16^, ^17^ There are also ongoing studies on different chemotherapy agents that could be incorporated in consolidation regimen for Ewing's sarcoma including doxorubicin, busulfan, mephalan, or even zoledronic acid.^18^, ^19^, ^20^

After 6 years of complete remission, our patient had an episode of collapse and was found to develop secondary brain metastasis. Brain metastasis is rare among children with solid tumors, usually presenting with seizure or hemiparesis.^21^ However, Ewing's sarcoma is the most common tumor causing secondary brain metastasis.^22^, ^23^ This might be due to the consequence of chemotherapy effectively suppressing systemic metastases while leaving the central nervous vulnerable due to blood‐brain barrier.^24^ A study of 335 Ewing's sarcoma patients shows that only 3.3% had brain metastases.^25^ Another study of 80 children with Ewing's sarcoma shows higher rate of 8.8% with secondary brain metastases.^21^ Due to the fact that Ewing's sarcoma is one of the commonest childhood tumor to develop brain metastases, prophylactic central nervous system irradiation and intrathecal methotrexate as part of initial therapy had been advocated in management of newly diagnosed Ewing's sarcoma in the past to prevent occurrence of central nervous system Ewing's sarcoma. However, the overall risk of secondary brain metastases was the same; hence, current regimens do not include these measures.^26^ The overall survival rate of secondary brain metastasis from Ewing's sarcoma is grave, usually with a period of 2 months from time of brain metastasis which improved to 7 months on surgical resection.^22^, ^27^, ^28^ However, two cases of long‐term survival were reported. They had combination of surgical resection, chemotherapy, and radiotherapy.^25^, ^29^ The literature on secondary brain metastases from Ewing's sarcoma is scarce. Most of them are focused on children with very minimal literature on young adults. This might be due to the fact that patients could be asymptomatic or present with minor symptoms such as headache.^30^ Further research or study is definitely required.

This paper puts forward a proposal for routine imaging such as MRI scan of head on follow‐up of patient with Ewing's sarcoma. Firstly, this might provide a more accurate understanding on the incidence of secondary brain metastases in Ewing's sarcoma. Secondly, this could help in diagnosing any brain metastases before the tumor grows large. Thirdly, this could improve the outcome by enabling surgical resection coupled with chemotherapy or radiotherapy when the tumor is small.

This case serves to improve our understanding toward the rare occurrence of secondary brain metastases of Ewing's sarcoma after long period of complete remission, as well as highlighting the importance of further study and research in this field. Routine brain imaging on follow‐up of patient with Ewing's sarcoma could be the next step forward in improving our understanding of this disease as well as improving long‐term outcome of patients.

**Learning points**

- Ewing's sarcoma is the most common childhood tumor to cause secondary brain metastasis.^22^, ^23^
- Surgical resection coupled with chemotherapy and radiotherapy provides the best long‐term outcome in management of primary or secondary Ewing's sarcoma.
- Routine brain imaging and long‐term follow‐up of Ewing's sarcoma could be the way to move forward by increasing our understanding in this area, as well as improving treatment and long‐term outcome for patients.

## CONFLICT OF INTEREST

The author of this study has no conflict of interest to declare.

## AUTHORS’ CONTRIBUTION

JZP: Conception and design, acquisition of data, analysis and interpretation of data, drafting of manuscript, and critical revision for important intellectual content.

## ETHICAL APPROVAL

No approval is required.

## Supporting information

App S1

## Data Availability

Data are available in article supplementary material.
