# Supplementary material for: Secondary brain metastases of Ewing's sarcoma presenting with collapse after 6 years of complete remission
Source: Clin Case Rep. 2020 Nov 24;9(1):560–5. doi: 10.1002/ccr3.3583 (PMC7813011; doi:10.1002/ccr3.3583)
Supplement: Supplementary file 1 — App S1 [file CCR3-9-560-s001.docx]

Appendix S1

I had a discussion with the patient regarding his thoughts on Ewing’s sarcoma, hoping that this would be helpful in understanding the case in a more holistic manner.

Patient’s Perspective

Having cancer as a sixteen-year-old is not only physically challenging, but emotionally and psychologically damaging to the welfare of the person suffering. When I was diagnosed with Ewing’s Sarcoma in 2013, my life crashed around me – my diagnosis came at the start of exam season, which would have been the start, at least to some extent, of adult life. But it also changed family life, day-to-day living and majorly effected my mobility and therefore independence.

The diagnosis was a shock, people always say “It’ll never happen to me”, but unfortunately science dictates that this is not indeed the case. My diagnosis marked the start of a year and a half of treatment, but a lifetime of worry and wariness of any small health problem.

To any person the prospect of the need for surgery is always going to be a worry. But the irony of cancer treatment is that this isn’t always the scariest part of the diagnosis. For me and for many others Chemotherapy is where the haunting nature of Cancer diagnosis comes. For within its grasps is nausea, tiredness, and other ailments that gather together to make suffering on daily basis – the new normal.

For me, the two chemotherapy regimes of VIDE and VIA in different parts of my treatment plan were where I struggled most. Both regimes would see me in hospital for days at a time for multiple sessions of the intoxicating medication. Personally, I find the word ‘medication’ Ironic, for as most people know, chemotherapy may make you better in the long term, but for the short term, it does completely the opposite.

Within hours of the first intravenous drip being put up, side-effects would start. Taste is perhaps the first hurdle, which may seem odd to state – my feelings are that many people experiencing chemotherapy regimes struggle with nausea not necessarily because of their stomach, but because of the overwhelming taste changes that occur when on Chemotherapy – especially when it is currently being infused.

For me, the taste was metallic but with an overwhelming chemical aftertaste that made eating incredibly difficult, all appetite was lost. The only thing that would decrease these side effects were strong tastes, incredibly sweet drinks, spicy food (something that I usually wouldn’t eat), and incredibly savoury foods such as Stuffing and Meat sandwiches.

But my taste affected me massively, it meant a reduced appetite and therefore a radically smaller intake of food, this did not help with treatment and meant that my strength was effected – this coupled with the nature of the diagnosis of a bone cancer, causing mobility issues within itself, meant that my ability to move around quickly or even have energy, in general, was low.

Other side effects which are more accurate and are very rarely mentioned in any accounts of the chemotherapy regime is the effects on bowel movements and urination. For me, chemotherapy meant that during and for many days after infusion my urine would have an overpoweringly strong smell that would send shivers down my spine and bring me close to vomiting. This would come coupled with excrement being of a very solid nature, and of a large size – which brought difficulty in making bowel movements and when they did come, going would be painful, with the feeling of ripping through the intestines. Whilst this seems detailed and somewhat disgusting it is the true nature of side-effects that are not usually mentioned. I would often find myself sat on the toilet for extended periods of time in order to pass movements that needed to be done – but be sat in pain for it to happen. The weird side would be that once this movement had passed, all subsequent movements would be normal.

Chemotherapy brings tiredness, which I was able to handle relatively well, I occupied my time awake with fun activities or going to school where possible. But when I needed sleep, I went. This was perhaps the easiest side effect to deal with.

Throughout the regime, there were many times that psychologically, I was not in a good place. At the age I was at diagnosis I was worried for my life, for my family and for the potential loss of future. But remaining focussed on goals by continuing at school helped me to stay positive and strong. It did not mean that there were not however events which were challenging. There were many questions asked by myself as to whether I could carry on with treatment which required many counselling sessions to ensure I remained positive with the mantra – “Positive Thoughts, Positive Outcomes”.

Of course, during this treatment plan, radiotherapy took a massive role, however, this was perhaps the easiest part of the process – with the taxing part being the commute to the hospital every day to be able to receive the treatment and fitting this around my schooling. Radiotherapy gave me some small side-effects such as sore skin in the target area, but in general, my overall energy levels went up, my personality started to show back through and it was seen as somewhat of a break in treatment in comparison to chemotherapy.

Surgery, on the other hand, was very invasive, requiring specialist care in Birmingham with an amazing team of orthopaedic surgeons. Difficult decisions had to be made by myself as to which surgery I should take – three options were given, one which may have left me with very minimal changes to my mobility but was high risk, one that would have seen the cancer removed but may have resulted in a collapse of the pelvis and therefore further surgery being required and finally the chosen surgery – a hemipelvectomy which would result in half of my pelvis being removed completely.

At a now seventeen years old, this kind of a decision was difficult, but with support from family and medical professionals, It was decided that the hemi-pelvectomy was the best course of action. It, however, meant a long road to mobility recovery, with a wheelchair being employed for many many months, alongside a rigorous physiotherapy regime to rehabilitate me into walking as independently as possible.

It took many years to come off crutches completely and whilst I require built-up shoes to walk today and I have a pronounced limp that is noticeable – for the most part my mobility is now unaffected. I am now independent and able to get around as required – which is a good outcome from what was an invasive surgery.

Throughout the entire treatment regime, pain medication was actively used – Ewing’s is invasive as the positioning on my pelvis putting pressure on nerves and other soft tissues meant that a plethora of different medications had to be employed in order to handle my pain. Whether it be Fentanyl patches, oxycodone, oxycontin or gabapentin, many different techniques were employed and cocktails of medications used at different times of treatment depending on how my pain changed over time.

All this comes to the conclusion however that I was young. This all happened at such a young age that I missed out on a lot of opportunities at the age that many of my friends were off enjoying themselves. Emotional trauma was perhaps the longest side effect – I now live in fear every day of it returning, a fear that was realised in 2020 when it did, unfortunately, return.

Cancer as a teenager is something I wish and hope to be eradicated. The effects are not just medical and health-related in terms of the obvious – but the damage to a person’s psychology is prominent and permanent. That being said I live every day and ensure that I take every opportunity that presents itself to see the world.

I hope that this information is supportive to someone and I would always be available to offer more information to those who request it.
